# The PDLIM family of actin-associated proteins and their emerging role in membrane trafficking

**DOI:** 10.1042/BST20220804

**Published:** 2023-12-14

**Authors:** Michael D. Healy, Brett M. Collins

**Affiliations:** The University of Queensland, Institute for Molecular Bioscience, St Lucia, Queensland 4072, Australia

**Keywords:** actin, commander, PDLIM, PDZ domain, SNX17, synaptopodin

## Abstract

The PDZ and LIM domain (PDLIM) proteins are associated with the actin cytoskeleton and have conserved in roles in metazoan actin organisation and function. They primarily function as scaffolds linking various proteins to actin and its binding partner α-actinin via two conserved domains; an N-terminal postsynaptic density 95, discs large and zonula occludens-1 (PDZ) domain, and either single or multiple C-terminal LIN-11, Isl-1 and MEC-3 (LIM) domains in the actinin-associated LIM protein (ALP)- and Enigma-related proteins, respectively. While their role in actin organisation, such as in stress fibres or in the Z-disc of muscle fibres is well known, emerging evidence also suggests a role in actin-dependent membrane trafficking in the endosomal system. This is mediated by a recently identified interaction with the sorting nexin 17 (SNX17) protein, an adaptor for the trafficking complex Commander which is itself intimately linked to actin-directed formation of endosomal recycling domains. In this review we focus on the currently understood structural basis for PDLIM function. The PDZ domains mediate direct binding to distinct classes of PDZ-binding motifs (PDZbms), including α-actinin and other actin-associated proteins, and a highly specific interaction with the type III PDZbm such as the one found in the C-terminus of SNX17. The structures of the LIM domains are less well characterised and how they engage with their ligands is completely unknown. Despite the lack of experimental structural data, we find that recently developed machine learning-based structure prediction methods provide insights into their potential interactions and provide a template for further studies of their molecular functions.

## Introduction

**Figure 1.**
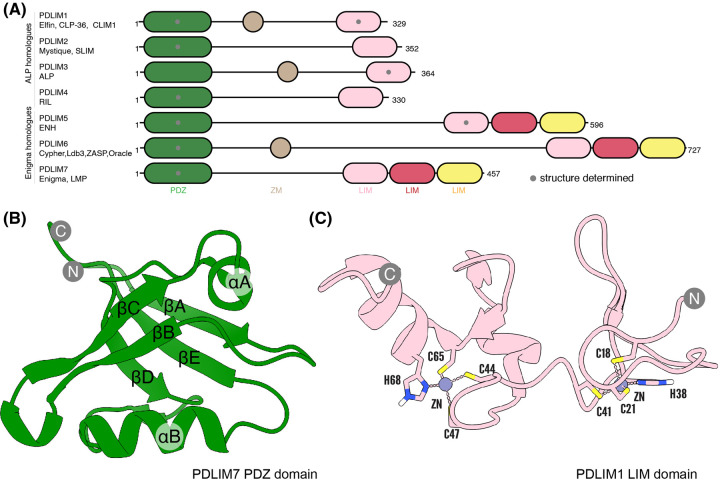
Structural overview of the PDLIM protein family. (**A**) Domain architecture of the PDLIM protein family (drawn to scale). The ALP homologues PDLIM1, PDLIM2, PDLIM3 and PDLIM4 contain a single LIM domain at their C-terminus while the Enigma homologues PDLIM5, PDLIM6 and PDLIM7 contain a triple tandem repeat LIM domain. Grey dots indicate domains for which experimental structures have been determined. (**B**) Example structure of the PDZ domain of PDLIM7 (PDB: 7RM8) [3]. The architecture of the PDZ domain is highly conserved containing five β-strands (βA-E) as well as one short (αA) and one long α-helix (αB). (**C**) Example structure of the LIM domain of PDLIM1 (PDB: 1X62). The LIM domains possess relatively divergent sequences overall, but contain two conserved zinc-binding motifs that stabilise their fold, with consensus sequence of Cx_2_Cx_16-23_Hx_2_Cx_2_Cx_2_Cx_16-21_Cx_2_[C/H/D] (where x denotes any residue).

The versatile PDLIM family plays many critical roles in actin organisation and function. There are seven members in humans, PDLIM1–7, with relatively broad tissue distribution apart from PDLIM6 which is exclusively expressed in muscle fibres [1]. The functional versatility of these proteins arises from the presence of both a PDZ (post synaptic density protein, *Drosophila* disc large and zonula occludens-1 protein) and LIM (Lin11, ISL-1, Mec-3) domain, which are prominent protein–protein interaction scaffolding domains (Figure 1A). Every PDLIM protein contains a single N-terminal PDZ domain, and can be subclassified based on the number of LIM domains. The actinin-associated LIM protein (ALP)-related proteins PDLIM1 (CLP-36), PDLIM2 (Mystique), PDLIM3 (ALP) and PDLIM4 (RIL) all contain only a single C-terminal LIM domain. The Enigma homologues are PDLIM5 (ENH), PDLIM6 (ZASP) and PDLIM7 (Enigma), which have a triple LIM domain. In both subtypes the PDZ and LIM domains are connected by a long (∼200 aa) intrinsically disordered linker.

PDLIM proteins are evolutionarily conserved [1,2]. Fischer and Schock [1] recently reviewed the evolution of the PDLIM protein family and concluded that the earliest PDLIM protein was found in Amphimedon in agreement with other work [3]. However, recent advances in structure prediction have now made it possible to search for proteins across species using both structure and sequence, and in preparing this review we used the FoldSeek server [4] and identified a putative PDLIM protein in the unicellular choanoflagellate *Salpingoeca rosetta* (Uniprot: F2UCV9). In all reported cases PDLIMs can associate with the actin cytoskeleton where they have roles in stress fibre repair [5–13] and Z-disc development [14–16]. This association with actin is driven by all elements of the PDLIM protein. The PDZ domain and the ZM motifs (a poorly defined motif found within the disordered linkers of PDLIM1, 3 and 6) have been shown to directly associate with α-actinin homologues [6], while the LIM domain has been shown to localise to actin through a conserved but unknown mechanism [12,13]. In addition to this many studies have also shown that the PDLIM PDZ domains are able to interact with other actin-associated molecules including YAP1 [17], Tropomyosin 2 [18] and Filamin A [19].

Several recent studies point to an important role for PDLIM proteins in intracellular membrane trafficking, particularly at the endosomal compartment. The endosome is a membrane-bound organelle that is a master regulator of transmembrane cargo sorting. In general cargo enters this organelle via anterograde transport from the Golgi, or from the cell surface following endocytosis. The endosome continues to mature to a multivesicular body, which fused with lysosomes where cargo is eventually degraded. Alternatively, cargo can be rescued from the maturing endosome and recycled back to the plasma membrane. This rescue is facilitated by a network of macromolecular assemblies that recognise short cytoplasmic sequences in transmembrane receptors and shuttle them back to the plasma membrane in tubulovesicular structures [20,21]. Recently direct interaction of PDLIM proteins was demonstrated with sorting nexin 17 (SNX17) [3], a protein critical for rescue of various cargos from the endosome [22,23]. SNX17 is recruited to the endosome through its PX domain which specifically interacts with the lipid phosphatidylinositol-3-phosphate (PI3P) [24]. The C-terminal FERM domain is then able to interact with transmembrane cargos that contain a NxxY motif (where x is any residue) [25–27]. This interaction subsequently leads to the recycling of this cargo in a poorly understood process that involves the sixteen-subunit Commander complex [28,29], as well as other machinery including Wiskott–Aldrich syndrome protein and SCAR homologue (WASH) complex which promotes formation of actin-rich membrane recycling domains [28]. Intriguingly, this cargo recycling can be blocked by a single point mutation in the carboxy-leucine of SNX17 (L470G) [3,28]. Recently, we used affinity mass spectrometry to show that a short peptide from the C-terminus of SNX17 is required for its binding to the Commander assembly. It was also discovered that the SNX17 C-terminus can mediate specific interactions with members of the actin-associated PDZ and LIM domain-containing (PDLIM) protein family [3].

In addition to their interactions with SNX17, many transmembrane proteins have been shown to depend on PDLIM family members for their correct localisation. This includes receptors such as the insulin receptor (INSR) [30], the tyrosine kinase RET [31], neurotrophin receptor p75 [32] and the α7 subunit of the nicotinic acetylcholine receptor [33] as well as cytoplasmic proteins such as NF-κB [34] and protein kinase C [35,36]. Taken together with the observation that PDLIM1 localises to the synaptosomes of chicken retina [37], and our own observation that a small pool of PDLIM5 and 7 colocalise with the EEA1-positive endosomes as well as the selective nature of the PDLIM PDZ domain for the type III PDZ-binding motif (PDZbm) found in SNX17 it suggests this protein family plays key roles in membrane trafficking pathways [3]. While the exact nature of this role is unclear at present it is worth noting that in 2004 Schulz et al. demonstrated that PDLIM4 could simultaneously interact with α-actinin, through its PDZ domain, and the cytoplasmic tail of the GluR-A subunit of the AMPA receptor, through the LIM domain [38]. They also observed that overexpression of PDLIM4 disrupted normal recycling of the AMPA receptor which led to accumulation within the dendritic spines [38]. This suggests that the presence of these two protein–protein interaction domains allows the PDLIM protein family to function as an adaptor or scaffold protein, coupling a given receptor to the actin cytoskeleton in a process critical for correct membrane localisation [39,40].

## PDLIM protein structure

While many proteins contain PDZ or LIM domains, it is the unique combination of these domains that defines the PDLIM family and leads to their diverse range of functions. Below we highlight some of the structural features of this family of proteins that contribute to their function and highlight several key interactors.

### The unique PDZ domain of PDLIM family members gives preferential interaction with type III PDZ motifs

The PDZ domain is perhaps one of the most well characterised protein–protein interaction scaffolds. With more than 267 examples found in more than 150 proteins in the human genome alone [41], this ancient structural motif is ∼80–100 residues in length and consists of five β-strands (βA-E) as well as one short (αA) and one long α-helix (αB) [42,43] (Figure 1B). Typically, these domains recognise short sequences at the C-terminus of interacting proteins called PDZ binding motifs (PDZbm) with binding affinities (*K*_d_) that fall within the micromolar range [3]. Broadly speaking these motifs involve the final three residues of a protein (with the final residue referred to as position 0) and can be classified into three distinct classes: class I, S/T-x-φ; class II, φ-x-φ; and class III, D/E-x-φ (where x is any residue and φ is any hydrophobic residue). While these final three residues are critical for binding, additional specificity is often imparted by other upstream residues in a given protein [44].

**Figure 2.**
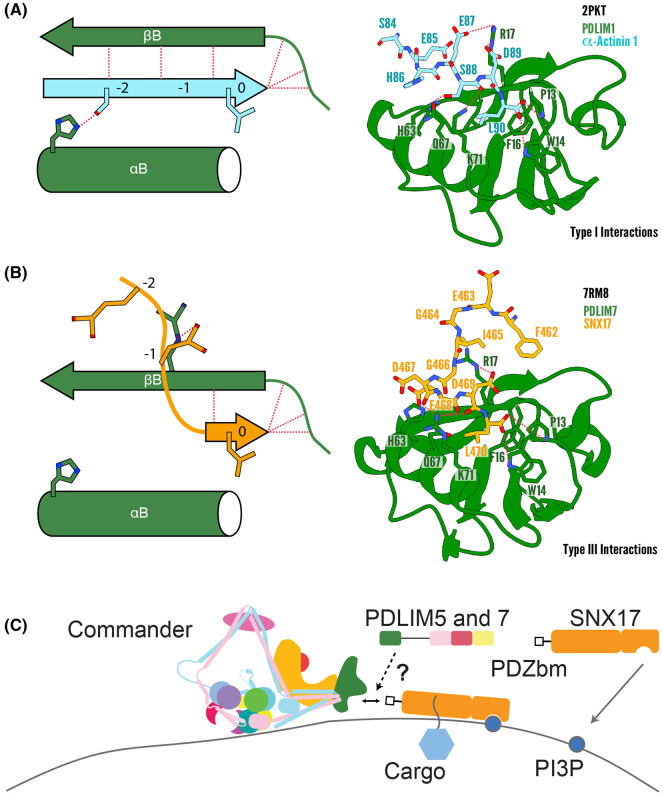
Binding of PDZbms to the PDLIM PDZ domains. (**A**) Canonical type I PDZbms interact with PDZ domains within the groove between the βB-strand and αB-helix, leading to the formation of a β-sheet augmentation. This interaction is stabilised by additional contacts made between the C-terminal carboxy group of the PDZbm and a conserved loop at the beginning of βB, and interaction of a Ser or Thr side chain at position −2 with a conserved His sidechain in the αB helix. This is shown schematically on the left, with an example on the right of the crystal structure of the PDZ domain of PDLIM1 bound to α-actinin-1 (PDB: 2PKT) [45]. (**B**) Unlike type I PDZbm type III PDZbm (as seen in SNX17) are unable to bind the traditional groove due to the steric hindrance from an acidic Glu sidechain in the −2 position. This pushes the peptide up and out of the groove. PDLIM proteins are uniquely suited to binding type III motifs due to the presence of a conserved Arg sidechain (Arg17 in PDLIM7) which stabilises this upright conformation. Again this is shown schematically on the left and with an example of the NMR structure of SNX17 bound to PDLIM7 (PDB: 7RM8) [3]. (**C**) Schematic representation of a potential pathway in which the PDLIM proteins act to mediate protein trafficking.

Conventionally, a PDZbm binds in the groove between the βB-strand and αB-helix, forming a β-sheet extension with its β-strand, with its C-terminal residue nested in the core binding pocket formed by the loop immediately preceding the βB-strand (Figure 2A). In the most common class I PDZbm (S/T-x-φ), the residue in the −2 position is a Ser or a Thr which makes a hydrogen bond to a highly conserved histidine in the αB helix. While this conventional interaction would still be achievable by class II PDZbms, the longer Asp and Glu sidechains present in class III PDZbm would be sterically precluded from the binding groove and unable to make the critical hydrogen bond with the conserved His sidechain.

Recently, using NMR spectroscopy we resolved the structure of the PDLIM7 PDZ domain bound to the type III PDZbm found in the C-terminus of SNX17 [3]. In this case the final residue Leu470 at position 0 was bound as expected, but instead of adopting the traditional ‘in groove’ binding the SNX17 peptide adopted an unconventional upright conformation. This is largely due to steric hindrance caused by Glu468 at position −2 in SNX17 which prevents association with His63 in PDLIM7, which forces the peptide to orient up and out of the conventional binding groove (Figure 2B). The loss of contact along the traditional groove is compensated for by the presence of an arginine (Arg17 in PDLIM7) that is conserved across the PDLIM family of proteins and contacts SNX17 Asp469 and Glu468 sidechains in the −1 and −2 positions, respectively. A similar binding mode was previously seen for the myotilin peptide (SEEL) bound to PDLIM6 where once again the residues in the −1 and −2 position make a complementary electrostatic interaction with Arg16 in PDLIM6 [45].

Another unique structural feature of the PDLIM PDZ domain is the carboxy-terminal binding loop which forms the core binding pocket. In virtually all PDZ domains there is a conserved glycine residue (Gly15 in PDLIM7) within this loop which provides main chain amides to facilitate binding to the carboxy residue. This glycine residue (referred to as residue ‘0’) is bookended by two hydrophobic residues in a xφGφ consensus sequence. PDLIM proteins invariably possess Pro, Trp, Phe residues in the −2, −1 and +1 positions, respectively. Mutation of the proline in PDLIM7 (P13G) abrogated SNX17 binding [3]. Intriguingly, the NMR spectra unambiguously shows this proline to be in the *cis* confirmation (PDB:7RM8) while the high resolution structure of myotilin bound to PDLIM6 PDZ (PDB:4YDP) shows the same proline in the *trans* confirmation, suggesting that this proline may be important to modulate loop flexibility and allow for a more buried C-terminal residue in the PDZbm. The combination of these conserved and unique structural features provides the PDLIM family of proteins with a platform for engagement of both type I and type III PDZbm such as those found in α-actinin and SNX17, respectively. Based on the studies of SNX17 and PDLIM proteins we proposed that the C-terminal PDZbm of SNX17 directs interactions with both the PDLIM proteins and the Commander complex itself [3] (Figure 2C), although the precise temporal and spatial regulation of these interactions remains to be determined.

### The LIM domains and their putative functions

The LIM domain is 40–60 residues in length and adopts a zinc finger fold to scaffold protein–protein interactions [46–49]. The typical consensus sequence for this domain is Cx_2_Cx_16-23_Hx_2_Cx_2_Cx_2_Cx_16-21_Cx_2_(C/H/D) (where x denotes any residue). The spacing of the conserved Cys and His residues allows the tetrahedral coordination of two Zn^2+^ ions that stabilises this structure [31,50] (Figure 1C). A large expansion of LIM domains occurred early on in eukaryote multicellularity, which also resulted in promiscuous incorporation of LIM domains into larger multidomain proteins; hence LIM domain-containing proteins have a wide variety of functions, many of which are related to the actin cytoskeleton [2,51]. More than half of the ∼70 LIM domain-containing proteins found in humans, including the PDLIM family of proteins, have been shown to be enriched at actin-rich domains such as focal adhesions, cell–cell adhesion sites and stress fibres [12,13,46,52].

Although it is well established that the LIM domain facilitates protein–protein interaction [50,53–55], unlike other domains, including the PDZ domain for example, there is no established consensus binding motif. This is true across the entire LIM domain family, and for the PDLIM proteins themselves. Early studies using a random peptide library identified that LIM3 of PDLIM7 bound to the endocytic signal peptide in the INSR (DGPLGPLYASSN) [30], while LIM2 interacted with the carboxy terminus of the receptor tyrosine kinase, RET (NKLY) [30,31]. The three LIM domains of PDLIM5 were also shown to interact with protein kinase C although no sequence mapping was conducted [35]. There are no published structures of PDLIM family LIM domains, but the NMR structures of the PDLIM1 and PDLIM3 single LIM domains and the first LIM domain of PDLIM5 have been determined by the RIKEN Structural Genomics Consortium (PDB IDs 1X62, 1X64 and 2DAR, respectively) (Figure 1C).

## Insights into PDLIM structure and function from Alphafold2

The recent development of the Alphafold2 algorithm has revolutionised the world of structural biology [56], allowing for predictions of over 200 million proteins with a high degree of confidence [57]. Thanks to implementations of this algorithm such as ColabFold [58] and RosettaFold2 [59] it is now possible to quickly and accurately predict the structure of an individual protein or large macromolecular assemblies [29,60–63]. In the final section of this review, we provide speculative insights into the PDLIM protein family derived from Alphafold2 predictions. Our intention is that these observations could warrant experimental validation and future functional investigations.

### LIM domain interactions

**Figure 3.**
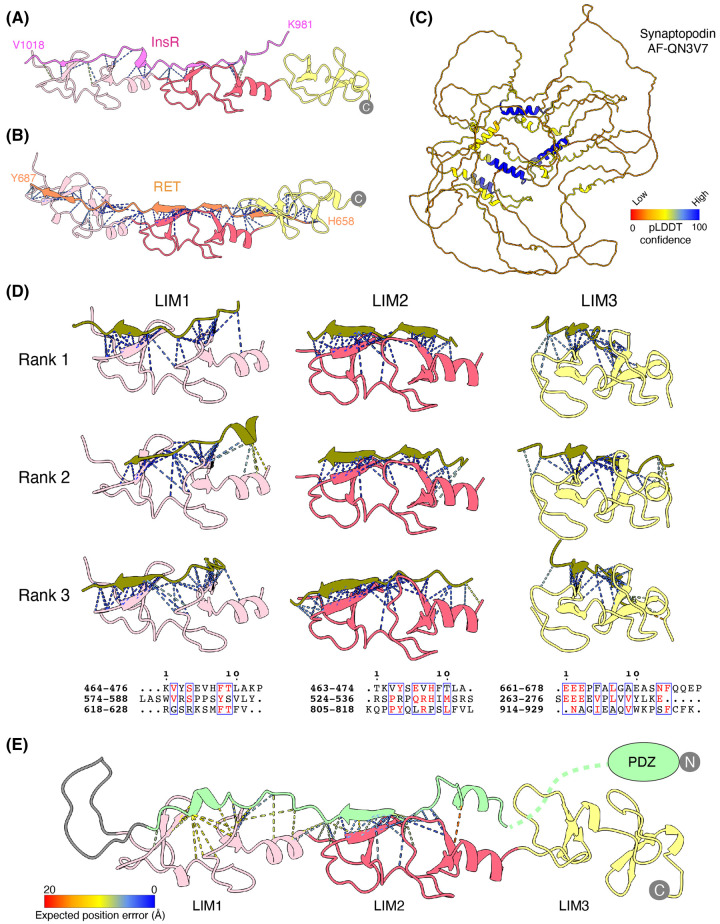
Insights from Alphafold: LIM domain interactions. (**A**) The cytoplasmic region of the insulin receptor (InsR) is predicted to make antiparallel contact with LIM1 and LIM2, while the tyrosine-protein kinase receptor RET (**B**) is predicted to interact with all three LIM domains. Dashed lines show predicted contacts within a 4 Å radius of any given residue and are coloured by expected position error (Å); a measure of confidence where red is low confidence, and blue is very confident. (**C**) Synaptopodin is predicted to be a largely disordered protein by Alphafold2. The structure is coloured by pLDDT score (AF-QN3V7). (**D**) Synaptopodin is predicted to interact with the three LIM domains of PDLIM7 using many diverse sequences. AlphaFold2 was used to predict PDLIM7 complexes with overlapping 50 aa fragments of synaptopodin, and almost the entirety of synaptopodin is confidently predicted to contact the three LIM domains using overlapping and divergent sequences. The top three ranked predicted LIM domain-synaptopodin complex are shown, ranked using the pDOCKQ score as previously described [72]. As in panel (**A**) dashed lines represent predicted interactions and are coloured by the expected position error. Below each LIM domain, the top three predicted sequences are shown. These generally display very low sequence similarity, suggesting that there are no clear ‘sequence motifs’ that are specifically recognised by each LIM domain. (**E**) The AlphaFold2 prediction of human PDLIM7. The disordered linker of PDLIM7 is predicted to form an intramolecular interaction encompassing both LIM1 and LIM2. As in panel (**A**) dashed lines represent predicted interactions and are coloured by the expected position error.

As mentioned above, much is known about the interactions between the PDZ domains and PDZbms, while very little is known about the mechanisms that underpin LIM domain interactions. To investigate putative LIM domain complexes we generated several predictions using AlphaFold2 implemented in ColabFold [58], beginning with the previously mentioned interactions of the PDLIM7 LIM domains with the InsR [30] and receptor tyrosine kinase RET [31] (Figure 3A,B). While the originally proposed InsR peptide showed only limited interactions with the LIM domains of PDLIM7, a longer sequence gave more confident predictions (^981^KRQPDGPLGPLYASSNPEYLSASDVFPCSVYVPDEWEV^1014^). In the case of RET, the C-terminal peptide (^658^HKFAHKPPISSAEMTFRRPAQAFPVSY^687^) is predicted to form a stable complex in an extended conformation that binds across all three of the LIM domains. The predictions provide support for these previous studies indicating that PDLIM proteins are able to directly engage with cargos through their LIM domains. Further work will be required to understand the diversity and specificity of these interactions.

Recent work investigating the spine apparatus, a specialised compartment of neuronal dendritic spines, identified a novel interaction between synaptopodin and PDLIM7 [64]. Synaptopodin is predicted to be largely unstructured (AlphaFold Protein Structure Database: AF-Q8N3V7-F1) (Figure 3C), and specifically localises to dendritic spines where it regulates actin dynamics by bundling actin in an α-actinin dependent manner [64,65]. Using Alphafold2 we performed a series of predictions of the structure of synaptopodin and fragments thereof in complex with PDLIM7. Intriguingly, this suggested that synaptopodin may be able to interact with the PDLIM7 LIM domains using many redundant and overlapping regions of its sequence (Figure 3D). Because multiple sequences in synaptopodin were predicted to interact with overlapping sites on the PDLIM7 LIM domains, it was necessary to run shorter sections to produce higher confidence predictions and more consistent models. Overall, these predictions suggest that regions across almost the entire synaptopodin protein can scaffold with the three PDLIM7 LIM domains. Very little sequence similarity is seen between these various structures suggesting (in agreement with previous work) that LIM domains are somewhat promiscuous in their protein scaffolding specificity (Figure 3D). Although synaptopodin and PDLIM7 show a high degree of co-localisation, PDLIM7 on its own is not strictly required for spine apparatus formation, potentially due to a high level of redundancy in actin-associated spine apparatus proteins [64]. The exact function of this interaction is, therefore, unknown at this point; however, it is likely that these sequence elements help to couple synaptopodin to actin filaments and create a molecular matrix for endoplasmic reticulum organisation in the spine apparatus [64].

### Disordered region of Enigma homologues is predicted to self-associate

Comparing the structures of the Enigma homologues PDLIM5–7 from the Alphafold database showed that the disordered region of these proteins may act as a self-associating intramolecular inhibitory peptide. Using PDLIM7 (AlphaFold Protein Structure Database: AF-Q9NR12-F1) as an example, it is predicted that a ∼25 aa stretch of the disordered region immediately upstream of the triple LIM domains forms a stable peptide interaction with LIM1 and LIM2 (Figure 3E). While the functional significance of this association is unclear it is reminiscent of the Zyxin self-associating peptide that prevents the Zyxin LIM domains from interacting with the actin modulating protein ZASP [66]. In that case the auto-inhibiting peptide is released by phosphorylation [67] allowing for regulated control of actin dynamics. Given that the PhosphoSitePlus database [68] reports many detected phosphorylation sites in the predicted self-associating sequences of PDLIM5–7 it is tempting to speculate that similar dynamics could regulate their function.

### Disordered regions of PDLIM proteins are predicted to bind α-actinin and actin

**Figure 4.**
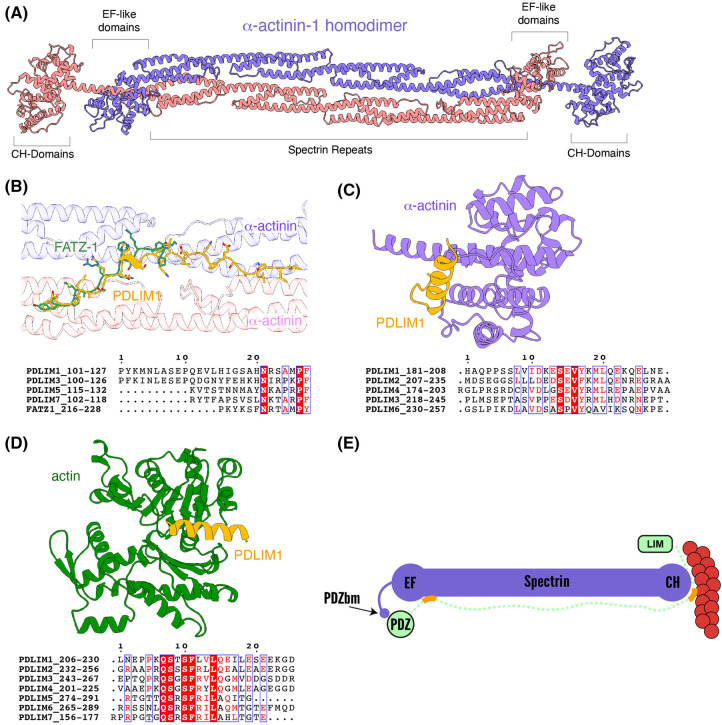
Insights from Alphafold: interactions of PDLIM proteins with α-actinin and actin. (**A**) α-actinin proteins form an antiparallel homodimeric complex through their spectrin repeats [69]. This positions the actin interacting CH domains at either end of the complex. (**B**) Using AlphaFold2, the PDLIM1 disordered domain is predicted to form an extended structure that interacts with the spectrin repeats of α-actinin-1. Overlaying this prediction with the structure of α-actinin-2 bound to the disordered region of FATZ-1 (PDB: 7A8T) [71] shows that they largely bind to the same surface. PDLIM1, PDLIM3, PDLIM5 and PDLIM7 are all predicted to interact with the spectrin repeats while PDLIM2, PDLIM4 and PDLIM6 show no predicted interaction (not shown). Alignment of the interacting sequences shows several conserved residues, pointing to a conserved binding motif to be validated by future biochemical and structural studies. (**C**) The disordered regions of PDLIM1, PDLIM2, PDLIM3, PDLIM4 and PDLIM6 are also predicted to possess sequences that interact with the CH-domain of α-actinin-1. The predicted structure of PDLIM1 is shown as an example and a sequence alignment shows many conserved residues. (**D**) Finally, Alphafold2 was used to predict if there is an interaction between the disordered domain of the PDLIM proteins and actin. Again, a representative structure between PDLIM1 and actin is shown, with several conserved binding residues seen in the sequence alignment. (**E**) Schematic representation of the PDLIM α-actinin-1 interaction.

The α-actinin1–4 proteins are perhaps the best-known interactors of the PDLIM protein family [5–10,17,19,36,38,65,69,70]. The α-actinin spectrin-like repeats form antiparallel homodimers positioning the N-terminal calponin homology (CH) domain and C-terminal calmodulin-like EF domains at either end [69,71] (Figure 4A). The N-terminal CH-domain is an actin binding domain [70] and thus through this homodimeric arrangement the α-actinins can act as cross-linkers of the actin cytoskeleton. While there is a well-established interaction between the α-actinin C-terminal type I PDZbm and the PDZ domains of the PDLIM protein family [45] (Figure 2A), recent work by Sponga *et al.* suggested that the disordered region of an α-actinin interacting protein called FATZ (filamin, α-actinin and telethonin-binding protein of the Z-disk, also known as myozenin-1 or calsarcin-2) forms a tight fuzzy complex that is critical for sarcomere function [71]. This prompted us to look more closely at the disordered regions of the PDLIM protein family. Indeed, we found that PDLIM1,3, 5 and 7 are all predicted to interact with the spectrin-like repeats of the α-actinin protein family via their disordered linker regions (Figure 4B). The region of the PDLIM proteins predicted to bind α-actinin show significant sequence and structural alignment with the previously resolved structure of α-actinin2 in complex with FATZ-1 (PDB: 7A8T) [71]. We also found two conserved regions of ∼20 amino acids in the disordered linker of the ALP-like subfamily of proteins (PDLIM1–4) that are predicted to, respectively, interact with both the actin binding CH domains of α-actinin and with actin itself (Figure 4C,D). These regions are just upstream of the LIM domains and would thus position the LIM domain near the actin cytoskeleton. These predictions, therefore, suggest that PDLIM proteins can engage in multiple reinforcing interactions with the α-actinin-associated actin cytoskeleton.

### Conclusion

The PDLIM proteins are a family of conserved protein–protein interaction scaffolds, connecting both membrane and cytosolic proteins to the actin cytoskeleton. Unique features in their PDZ domains make them ideally suited to bind not only the more common type I PDZbm but also to type III PDZ motifs such as the one recently identified in the SNX17 endosomal trafficking adaptor [3]. The observation that perturbation of PDLIM family members leads to redistribution of various receptors (e.g. the AMPA receptor [38]) suggests these proteins may play key roles in regulation of membrane trafficking, acting as bridges between cargo, trafficking complexes and the actin cytoskeleton. Although structural details on the LIM domains remain limited, predictions outlined here suggest they might mediate direct interactions with a variety of peptide sequences in transmembrane ligands such as the InsR and RET [30,31] and cytoplasmic membrane organisers such as synaptopodin [64]. Furthermore, the disordered linkers in PDLIM proteins themselves appear to also mediate intramolecular interactions with their LIM domains, as well as intermolecular association with α-actinin and actin proteins. While these predictions now require experimental verification, they point to a highly regulated and broadly distributed scaffolding capability across their entire sequences.

## Perspectives

- PDLIM proteins are critical for organisation of the actin cytoskeleton and mediating the association of many different cytosolic and membrane-bound proteins with actin fibres. Recent work points to an important role in the cellular localisation of a variety of transmembrane proteins and a functional connection to endosomal trafficking complexes such as SNX17 and Commander.
- The PDLIM protein family act via co-ordinated protein scaffolding mediated by their three key domains: the N-terminal PDZ domain, C-terminal LIM domain(s) and a long central unstructured linker. Our structural understanding of how the PDZ domain associates with C-terminal PDZbms is extensive, and it is known that key features of the PDLIM PDZ domains allow for binding of both canonical type I binding motifs, and specific coordination of type III binding motifs such as the one possessed by SNX17. The LIM domains remain poorly characterised at the structural level, but predictions using the AlphaFold2 machine learning-based method suggest that some family members can mediate both intramolecular interactions with their own unstructured linker as well as binding to extended peptide sequences in both cytosolic and transmembrane proteins. Finally, the linker regions of the PDLIM proteins are also predicted to mediate direct interactions with actin itself and the actin-cross-linking α-actinin proteins.
- Many unanswered questions remain regarding the molecular basis for PDLIM activity. Predictions provide a basis for generating hypotheses regarding the mechanism of PDLIM function but require further experimental validation. Whether PDLIM proteins are regulated by intramolecular interactions and undergo conformational changes is also an important issue to address.

## Open Access

Open access for this article was enabled by the participation of University of Queensland in an all-inclusive *Read & Publish* agreement with Portland Press and the Biochemical Society under a transformative agreement with CAUL.

## Abbreviations

ALP: actinin-associated LIM protein
CH: calponin homology
FATZ: filamin, α-actinin and telethonin-binding protein of the Z-disk
INSR: insulin receptor
LIM: LIN-11, Isl-1 and MEC-3
PDLIM: PDZ and LIM domain
PDZbms: PDZ-binding motifs
SNX17: sorting nexin 17
